# The Special Features of Prenatal and Preimplantation Genetic Counseling in Arab Countries

**DOI:** 10.3390/genes13020167

**Published:** 2022-01-18

**Authors:** Shaza D. Malik, Mashael Al-Shafai, Atiyeh M. Abdallah

**Affiliations:** Department of Biomedical Sciences, College of Health Sciences, QU Health, Qatar University, Doha 2713, Qatar; shazamalik90@yahoo.com (S.D.M.); malshafai@qu.edu.qa (M.A.-S.)

**Keywords:** prenatal diagnosis, Arab, consanguinity, genetic counseling, preimplantation genetic diagnosis

## Abstract

Genetic counseling services have only recently been introduced in most Arab countries, and their utilization is increasing. Prenatal genetic counseling is essential, particularly in the Arab context, which is characterized by high rates of consanguinity. Nevertheless, little is known about the decisions faced by parents and the factors underlying the complex decision making that must occur when accessing these services in Arab countries. Herein, we performed a narrative review to discuss the reported experiences of parents accessing genetic counseling in the prenatal setting in the 22 Arab countries. We also highlight the different types of decisions encountered and the factors influencing them. We report that: (i) utilization of genetic counseling services varies across different Arab countries; (ii) many factors affect decision making and service utilization, especially religion; and (iii) parents are faced with an array of decisions in the prenatal setting, partly driven by increased utilization of prenatal diagnosis and preimplantation genetic testing in some countries. Our work is the first to highlight the different factors and decisions influencing genetic counseling in Arab countries. Understanding these factors is essential for improving genetic counseling services in the region and helping counselors facilitate informed decision making.

## 1. Introduction

The characterization of the “Arab genome” has renewed scientific interest in its unique features and complexities [1]. Up to 50% of all marriages in the Arab world are consanguineous due to cultural, social, and political norms [2,3]. Consanguinity poses a risk factor for genetic diseases because it increases the risk of inheriting autosomal recessive disorders through shared genetic material from common ancestors. Prenatal, pediatric, premarital, and cancer genetic counseling are important in any part of the world [4]. However, in the Arab world, prenatal genetic counseling is particularly important, as it can reduce the otherwise high incidence of genetic diseases [5]. The ultimate goal of prenatal genetic counseling is to allow couples to make informed decisions regarding current or possible future pregnancies. This is achieved through prenatal screening or diagnostic genetic testing. Screening testing, such as second-trimester maternal serum α fetoprotein (AFP) and ultrasound, are offered to all pregnant women [6]. Diagnostic genetic testing is offered to women with high-risk pregnancies, including those with a family history of a particular genetic disease, advanced maternal age, or positive sonographic test results, as well as when screening results are positive [7].

Genetic counseling is a core part of the management of families with suspected or confirmed genetic conditions [8]. Many of the 22 Arab countries have no available literature on the implementation of genetic counseling services, including from Djibouti, Libya, Mauritania, Somalia, and Sudan. However, some of the Gulf Cooperation Council countries and other Arab countries do offer these services (Table 1) [9,10,11,12,13,14,15,16]. The implementation of services in those countries is probably due to the financial means of those countries, high consanguinity rates driving a high incidence of some genetic diseases, as well as the availability of genetic counselors [11,17,18]. Other Arab countries have demonstrated a need for genetic counseling services; however, they face significant barriers to implementation of said services due to poor healthcare infrastructure, a lack of resources and shortage of genetic counselors, the high cost of genetic testing, poor public knowledge about the risk of genetic diseases, and limited access to healthcare services [19,20,21,22]. These disparities have led to variability in the type and quality of provided genetic services in different countries [23]. Another advance that has broadened the scope of prenatal genetic counseling is the increasing availability of in vitro fertilization (IVF) coupled with preimplantation genetic testing (PGT). PGT is used when parents are confirmed (molecularly) to be carriers of pathogenic variants associated with known genetic conditions or they are affected themselves. This allows for the identification of embryos carrying the inherited genetic defects and selecting against them when appropriate [24]. There are two main types of PGT: PGT for aneuploidy screening (PGT-A) and PGT for monogenic disease screening (PGT-M). PGT use has increased over the past 20 years due to technological advances and decreased costs [25]. The acceptance of PGT is associated with many factors, including socioeconomic status, religion, epidemiological determinants, and rates of autosomal recessive conditions [26]. In prenatal counseling sessions, counselors might offer different options, including prenatal screening tests, diagnostic tests, PGT, and termination of pregnancy (TOP) [27,28], when relevant.

Prenatal genetic counseling services have therefore been implemented in some Arab countries. There is relatively little evidence concerning the factors affecting delivery, evaluation, and outcomes of genetic counseling or concerning user and counselor experiences of these services in the Arab region. We therefore reviewed the types of decisions and the factors affecting decision making in the prenatal setting in the Arab context. Box 1 summarizes our search methodology and Box 2 summarizes the main outcomes from this review.

Box 1 **Search strategy and selection criteria.**

Two independent searches of the PubMed, Embase, Scopus, and Cochrane Li-brary databases were conducted between October 2020 and December 2020. The first search included the following terms: “genetic counseling” AND “Arab/middle east” AND “preimplantation” OR “PGT” OR “PGT”. The second search included the combination of the following terms: “genetic counseling” AND “Arab” AND “pre-natal”. Only papers published in English were reviewed. The final reference list was collected based on originality and relevance to the scope of this review.

## 2. Consanguinity and the Need for Prenatal Counseling

Genetic counselors are needed in every health system due to their integral role in increasing knowledge and awareness about hereditary conditions in high-risk/affected individuals, their mode of inheritance, and management options. Any information provided ultimately helps patients to make informed decisions. The Arab region is characterized by large families and high consanguinity rates, with first-cousin marriages being the most common. For example, in the Kingdom of Saudi Arabia (KSA), the rate of consanguinity is 57.7%, with first-cousin marriages being the most common form (28.4%) [29]. Consanguinity drives the vital need for prenatal genetic counseling in many Arab countries, where a significant proportion of fetal morbidity and mortality is linked to genetic conditions [30]. Furthermore, consanguinity is associated with adult-onset genetic disorders and some multifactorial diseases [31]. Genetic counseling thus represents a form of management that reduces the incidence of genetic conditions. Counselors help family planning through facilitating informed decision making. They do this by providing information about recurrent risk and reproductive options, as well as genetic testing and its limitations [32].

In no small part due to consanguinity, the Arab region is characterized by a high incidence of genetic and congenital disease and hemoglobinopathies [30]. Some of those genetic diseases are associated with variable levels of penetrance and expressivity. These are often difficult concepts for people to understand, meaning that the educational role of genetic counselors is both integral and complicated [33]. Moreover, there is an even greater need for genetic counseling because genetic diseases are often stigmatized in Arab countries [9]. For example, people who pursue genetic carrier testing for familial conditions feel that this testing divides the society, creating different social layers within a population [34]. Therefore, genetic counselors needs to explain such misunderstandings to help patients make informed choices that work best for them [35]. In Qatar and KSA, where there are established genetic testing services; counselors play a particularly critical role in family planning based on the results of genetic testing and the classical scope of genetic counseling services [32]. Generally, the need for prenatal services increases when partners are carriers of the same genetic diseases, a situation that is more common in consanguineous populations [36].

Box 2 **Key points.**

**What is known about this topic**

- There is abundant literature on the factors affecting decision making and the types of decisions made by parents accessing prenatal genetic counseling ser-vices around the world.
- Most of the 22 Arab countries still do not provide genetic counseling services, despite a significant need, given the elevated consanguinity rates in the region.
- It is known that social and cultural factors influence decision making around access to and uptake of prenatal genetic counseling services, although the fac-tors specific to the Arab world are less well documented.

**What this paper adds to the topic**

- Utilization of genetic counseling services varies across different Arab countries.
- Many factors affect decision making and service utilization in the region, not least religion.
- Knowledge of these factors, especially religious drivers of acceptance of prena-tal genetic counseling services, can help to improve informed decision making in the Arab world and beyond.

## 3. The Decisions Faced in Prenatal Settings

Prenatal options vary based on several factors, including the stage of family planning (prior to or during pregnancy) and the existence of a molecular diagnosis in the parents. Nevertheless, these options can be classified into four general themes. The first is prenatal genetic screening, such as non-invasive prenatal testing (NIPT), which is used in the following circumstances: high-risk pregnancies, abnormal fetal ultrasound findings and positive family history [37]. Second is prenatal diagnosis or testing, which is utilized when screening tests suggest abnormalities in the current pregnancy. This can be achieved through different tests, such as karyotyping, single-gene testing, gene panels, or whole-exome sequencing [38]. Third is IVF with PGT, which may be considered when one or both parents are confirmed (molecularly) as affected by or carriers of a genetic disease. This is usually used when couples are planning a future pregnancy [39]. Finally, TOP is used when the current pregnancy is affected, whether genetically confirmed or not and regardless of the parents’ carrier status [40,41]. Each of these themes has different implications and consequences for family and its dynamics.

A screening test, NIPT, offers the significant advantage of avoiding miscarriage risk associated with invasive diagnostic procedures, such as chorionic villus sampling (CVS) and amniocentesis [42]. In NIPT, fetal DNA is extracted from maternal blood samples for subsequent genetic testing. It can be performed as early as nine weeks of pregnancy [43]. NIPT has a sensitivity of 96–100% and a specificity of 94–99.9% for detecting chromosomal aneuploidies, including trisomies 21, 18, and 13 [44] and might aid in decision making regarding early TOP in affected individuals. There are ethical issue surrounding considering TOP after positive NIPT results because the test is associated with a relatively high false-positive rate. This is because the sample collected for NIPT is of placental rather than fetal origin. For example, detected aneuploidy might be presented in the placenta but not the fetus [45]. Nevertheless, NIPT might help approve TOP according to Islamic guidelines through early detection (before ensoulment; see below) [46]. Furthermore, other standard screening tests exist, such as maternal serum screening [47].

In the Arab context, the option of not having children is not always considered when parents are carriers or affected by a genetic condition. Having children is integral in family dynamics [48], especially in consanguineous marriages, in which having children is perceived as a way of strengthening family bonds, as large families are a source of pride [49]. Instead of not having children, couples consider prenatal genetic testing or IVF-PGT as more appropriate options to avoid having an affected child [50]. Despite resistance to not having children, a cohort of Israeli Arab parents consider having an affected child as unfair [51]. Therefore, there is acceptance that genetic counseling helps to reduce the risk of having an affected child through genetic screening and testing [51,52].

PGT usually results in successful pregnancy outcomes and is a convenient choice for people with a religious opposition to TOP. PGT is used to select healthy non-carrier embryos and, if not available, healthy carrier embryos, delivering an embedded risk of 4% of a carrier to be affected [53]. In KSA, PGT is allowed for severe genetic conditions, such as trisomy 13, and is accepted from a religious perspective. However, genetic counseling services for couples who undergo PGT are still not common in KSA [54]. The decision to undergo TOP is frequently encountered in the prenatal setting. Accordingly, many people opposed to TOP might decline prenatal counseling [55]. Attitudes towards TOP appear to be variable, with 42% of parents indicating that they might select TOP, 12% refusing the option, and 8.5% indicating uncertainty, although parents reported that having a sick child was a legitimate reason to consider TOP [56]. Moreover, TOP rates are variable in different Arab countries. In Tunisia, for instance, 94.7% of individuals selected TOP after being diagnosed with a serious genetic condition [57].

A cohort of Saudi students reported contradicting attitudes when TOP was discussed as an option for untreatable genetic conditions [58]. However, compared with previous reports from KSA, a greater percentage of couples (~50%) considered discussing abortion, compared with ≤26% in older reports [59,60]. A narrative review discussing the Arab countries presented a range of reasons why TOP would be considered legitimate. An average of 98% of Arab countries consider saving the mother’s life a legitimate reason for TOP. However, only 33% of Arabs consider social and financial reasons as legitimate [61].

The decision to undergo TOP depends on the time of diagnosis. This is a crucial factor in decision making and highlights the importance of early genetic counseling for at-risk couples conceiving a child [62]. In a cohort of Bedouin Arabs, TOP was no longer an option in ~30% of the sample due to late gestational age [63]. Since the option to undergo TOP is linked to Muslim laws, it is not available in many cases. However, PGT has partially resolved this issue, for instance, by allowing genetic testing to be conducted before implantation. In Oman, couples who underwent PGT reported that the experience was physically and emotionally tiring [64]. Among Israeli Arab women, the decision to terminate a pregnancy was not different in women receiving counseling compared to those who did not [65]. These data highlight deeply rooted cultural and religious beliefs and the consequent difficulties faced by counselors when discussing topics such as TOP. The increased incidence of genetic conditions in some Arab counties might also be attributable to a lack of services offering TOP. PGT might therefore be a suitable reproductive alternative even though a majority of couples reported anxiety as a dominant feeling throughout the process [66].

In a study assessing the acceptance of TOP among Arab Muslim females with fetuses affected by congenital anomalies, TOP was not offered in all cases [67]. Females who chose to continue the pregnancy reported emotional attachment. Physicians and healthcare providers showed understanding of this feeling when delivering risk information. In a study of PGT acceptance as an alternative to prenatal diagnosis in Lebanese couples, 68% of all participants indicated a preference for PGT because it helps to avoid TOP and reduces the stress associated with waiting for the results of prenatal diagnosis [68]. Among those participants, 100% of females with a previous history of termination chose PGT as an alternative. In a study comparing PGT and prenatal diagnosis in Saudi couples, there were no significant differences between the preferences for any of the two options; however, in couples who disagreed on which procedure to opt for, more females preferred PGT over prenatal diagnosis than males [69]. Among pregnant Israeli Arabs, decision making around TOP was dependent on many factors, including religion, level of education, age, and having had a previous experience with the relevant condition, whereas TOP was preferred among parents who were educated, younger, and had previous experience with an affected child [56]. Furthermore, attitudes towards TOP were variable, with 42% of parents indicating that they might select TOP, 12% refusing the option, and 8.5% indicating uncertainty. In addition, 42.2% of parents reported that having a sick child was a legitimate reason to consider TOP.

## 4. Factors Affecting the Acceptance of Prenatal Genetic Counseling Services

In the Arab world, there are many barriers to the acceptance and delivery of prenatal genetic counseling services. These include lack of national databases, scarcity of population-specific genetic information, relatively new service provision, moderate experience in delivering those services, ethical and legal considerations, and the availability of testing and interpretation of test results [70,71]. Generally, factors associated with public acceptance are determined by cultural, social, and religious factors, which are discussed more fully in the Arab context below.

### 4.1. Social Factors

Social factors, such as socioeconomic status, health insurance, and accessibility of the service, are known to influence the uptake of genetic counseling services [72,73,74]. For example, among pregnant Israeli Arabs, several factors were associated with the acceptance of prenatal counseling services. First, low financial affluence was associated with service rejection due to its high cost. This finding was replicated in a cohort of Tunisian couples among whom service acceptance was dependent on socioeconomic status [57]. Additionally, among pregnant Israeli Arab women, service utilization was lower among housewives [75]. In another example, service accessibility can be impacted by transport availability, particularly in rural areas [76]. In Jordan, the cost of genetic testing and genetic counseling negatively impacted the utilization of those services due to the fact that the service is still new and yet not implemented in a way that is cost-effective [77].

### 4.2. Religious Factors

Islam is the most common religion in the Arab world, so it is essential to consider how Islamic views tackle some of the issues arising in the setting of prenatal services. The most obvious example is decisions related to TOP, in which couples take into consideration the Islamic Fatwa. This states that TOP can be considered before 120 days from gestation, i.e., before ensoulment, and for severe conditions only [78]. Some advances, such as assisted reproductive technology, are relatively new, and they may have not been discussed in relation to Islamic Sharia Law; therefore, they might raise ethical questions among Muslims [79]. Addressing new advances from the Islamic perspective under Sharia Law requires consideration of Islam’s primary and secondary resources. Primary resources include the Quran and the practices of the prophet and the Hadith, thereby providing adaptation to new emerging issues [80,81]. However, Islam generally advocates saving human life and protecting it by all available means; accordingly, Islamic scholars and jurists accept some of the most common options in the prenatal setting, such as prenatal diagnosis and PGT [82].

The literature seems to suggest that in Arab countries, religion—particularly Islam—is a common factor affecting decision making and utilization of genetic services. Therefore, Islamic beliefs must be considered when counseling Muslim couples or communities, particularly when discussing reproductive options, in order to better understand them, provide legitimacy, and support decision making [83]. Moreover, for proper integration of religion into counseling sessions, genetic counselors must have two levels of understanding about Islam: theological and ethical. A theological understanding is achieved by learning about concepts of faith and destiny in Islam, while an ethical understanding is achieved by learning about both general ethical principles and those specific to Islam [83].

Religious beliefs are also a significant factor affecting service utilization and decision making. Among pregnant Israeli Arabs, 92.7% of women view themselves as religious, and 40% of that cohort of women do not favor attending genetic services because they believe that counselors would be dismissive of their religious beliefs [76]. In KSA, a key factor that changed the attitudes towards acceptance of prenatal diagnosis and TOP was education about Islamic Fatwa on TOP. Patients were educated about the period during which TOP is accepted in Islam (prior to 120 days) [84], as well as assisted reproductive technology [85]. In addition, couples reporting lower levels of religious beliefs had increased utilization of prenatal services [86]. In Egypt, when pregnant females were properly counseled on religious aspects addressing Islamic views on prenatal diagnosis and TOP, 100% of females with a fetus affected by thalassemia chose TOP, reflecting the importance of taking the region’s beliefs into account in shaping patients’ attitudes towards TOP [87]. A Tunisian study reported similar outcomes, in which 94.7% of all pregnant Muslim women with abnormal genetic results chose to undergo TOP. This was attributed to the quality of information provided by the genetic counselor, which helped patients to understand this option [57].

### 4.3. Cultural Factors

Culture is another critical factor that affects decision making. In a study comparing perceptions about prenatal genetic counseling between native Palestinians and American Palestinians, differences were noted across many levels. American Palestinians perceived genetic counselors as having a nondirective approach and favoring female choice concerning prenatal options in the face of disagreements between couples. By contrast, native Palestinians viewed the counselor as having a directive approach and favored male decision making [88]. Another factor that might decrease service utilization is the fear of stigmatization and being labeled as having a genetic condition. In a cohort of Omani patients, fear of stigmatization was an essential factor, particularly for females, since they are more socially affected than men [89]. Similar findings were noted in KSA, where participants reported stigmatization as a factor discouraging them from seeking prenatal genetic counseling. Counseling resulted in a change in perception about genetic diseases and associated stigmatization [90].

### 4.4. Miscellaneous Factors

Some factors, such as the education level of the parents, have only been discussed in a few countries. For example, in Palestine, educated parents had greater knowledge about genetic disorders, which resulted in them having a positive attitude toward the genetic counselor [15]. The impact of the type and severity of disease on service utilization has also been reported; attitudes towards prenatal genetic testing and different options vary depending on the severity of the condition. In a cohort of Israeli Arab parents, attitudes towards genetic counseling were significantly in favor of TOP for severe diseases due to the perception that it is unfair to have a child affected by severe disease. Meanwhile, they were less in favor of TOP for other “milder” conditions, such as trisomy 21 [51]. Another less commonly discussed factor is the impact of partners’ opinions and the impact of the extended family of partners. For example, among pregnant Israeli Arab women, service utilization was lower among women whose partners opposed TOP [75]. In countries where Arabs are considered minorities, genetic services are generally less accessible, resulting in a negative attitude towards genetic services and a misunderstanding of the service scope, thereby decreasing service utilization [91]. In addition, the perception of the service and its implications for health are factors associated with decreased utilization. For example, among pregnant Israeli Arab women, females who perceived the prenatal procedures as a risk factor for miscarriage did not agree to undergo prenatal diagnosis [75]. On the other hand, positive attitudes towards counseling and the service were associated with higher acceptance [76]. In addition, in a study assessing the acceptance of prenatal diagnosis among Israeli Arab women, many had misperceptions about prenatal diagnosis, with ~50% of women believing that prenatal diagnosis could not accurately diagnose fetuses with malformations or anomalies; only 22% believed that it could accurately diagnose affected fetuses. Despite that, 95% of females in the study indicated their willingness to undergo prenatal diagnosis if it was requested [92]. This highlights a key issue that might influence genetic counseling and its uptake in the region, which is a misunderstanding surrounding the non-directive nature of the profession, given that many Arab patients are more accustomed to a directive form of medicine.

**Figure 1:**
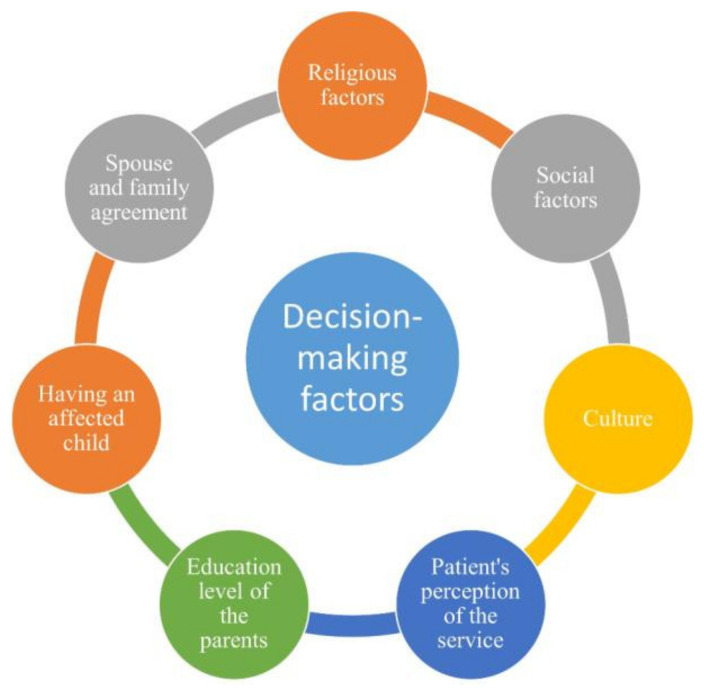
The different factors affecting decision making in prenatal genetic clinics among Arab parents.

Other factors that cause low utilization of PGT include its high cost, lack of knowledge about the technology, and overall lack of information regarding reproductive options for couples [86,93,94]. In KSA, couples were willing to undergo PGT due to a wish to have a healthy child. The majority of couples with at least one affected child showed that the technical limitations of PGT were not a concern [95]. Similar findings were also obtained in a separate study targeting the same population, in which the most important factors affecting parental decision making were the number of affected children, the associated burden of raising them, the availability of prenatal genetic testing, and availability of TOP [90]. In one study from Tunisia, despite genetic counseling providing all possible reproductive options to couples, many refused prenatal testing. This occurred despite an overall positive perception of the service in the country [16]. In Jordan, around 30% of participants indicated that genetic counseling is an essential service for people with genetic conditions and that their role in education is vital for decision making. This highlights the importance of information provided by the genetic counselor in assisting the decision making process [77]. Figure 1 shows the various overall factors affecting Arab patients’ decision making in the counseling clinic.

## 5. Genetic Counseling Service in Qatar

There has been very little research exploring experiences of prenatal genetic counseling services from a patient perspective in Qatar. However, based on the literature and sociocultural similarities, Qatar is likely to share the same ethical and legal problems concerning prenatal services as other CGG countries, for example, the window of legal abortion (prior to 19 weeks of gestation). Prenatal genetic counseling services in Qatar are provided in the Women’s Wellness and Research Center, which belongs to Hamad Medical Corporation (HMC). The service is managed by the Medical Genetics Department of HMC. Referrals to prenatal genetic counseling services are made for several reasons, including a positive family history for a genetic disease, abnormal fetal ultrasound, abnormal screening results, and advanced maternal age.

In the clinic, it is usually preferable to see couples (rather than either parent alone) to ensure that a proper and equal amount of information is delivered to both parents, which can help them to reach a mutually agreed-upon decision. For example, the decision to undergo TOP might depend on the religious adherence of the couple and the severity of the condition. Prenatal genetic counseling services in Qatar are growing, as reflected by the enormous number of referrals made to HMC, the main healthcare-providing institution in Qatar. In prenatal genetic counseling sessions, patients experience different emotions when receiving a diagnosis of a genetic disease, ranging from acceptance of the diagnosis to denial. The counselor needs to have the necessary skills to address these varied and often difficult emotions.

## 6. Conclusions

Offering a prenatal genetic diagnosis or PGT is vital for reducing the incidence of genetic conditions. One of the most important roles for the counselor is to facilitate decision making. There is currently a lack of evidence discussing genetic counseling from the legal and ethical perspective in the region. Nevertheless, the factors discussed in this review should help counselors in the region to maintain cultural sensitivity by being aware of the couple’s motivating factors and critical concerns. Decisions faced in the prenatal setting are variable and have different implications for the individual, family, and society. Thus, the role of genetic counselors is particularly critical and sensitive when facilitating decision making. Further work is needed to explore counselors’ perspectives in this region.

We recommend that counselors in the region start to ask patients for feedback after the session. This can be achieved by providing patients with a questionnaire to assess, for example, patient satisfaction about the quality and quantity of information supplied, opinions about the delivered information, issues that should be discussed in future sessions, and patients’ willingness to discuss religion and its implications for service provision. Indeed, engaging religious scholars, such as imams and rabbis, to address some misconceptions related to prenatal genetic services and other healthcare issues has been implemented in the Western world [96,97]. This feedback might help counselors improve the quality of the session and help patients by explicitly meeting their needs and demands. We also believe that more research should be conducted to explore the quality and experience of genetic counseling in the region from the perspective of both patients and counselors.

## Figures and Tables

**Table 1:** The implementation status of genetic counseling services across the 22 Arab countries based on the available literature.

Countries with Established Genetic Counseling Services	Countries Demonstrating a Need for Genetic Counseling Services	Countries with No Reported Genetic Counseling Services
Saudi Arabia	Algeria	Djibouti
Bahrain	Iraq	Libya
Egypt	Yemen	Mauritania
Jordan		Somalia
Lebanon		Sudan
Morocco		Comoros
Oman		
Qatar		
Palestine		
Tunisia		
United Arab Emirates Kuwait		
